# Efficacy and Safety of Direct Oral Anticoagulants in Patients With Atrial Fibrillation and High Thromboembolic Risk. A Systematic Review

**DOI:** 10.3389/fphar.2019.01048

**Published:** 2019-09-19

**Authors:** Domenico Acanfora, Marco Matteo Ciccone, Pietro Scicchitano, Giovanni Ricci, Chiara Acanfora, Massimo Uguccioni, Gerardo Casucci

**Affiliations:** ^1^Unit of Internal Medicine, San Francesco Hospital, Telese Terme, Italy; ^2^Section of Cardiovascular Diseases, Department of Emergency and Organ Transplantation, School of Medicine, University of Bari, Bari, Italy; ^3^Cardiology Unit, Hospital “F. Perinei” Altamura (BA)–ASL BA, Bari, Italy; ^4^Unit of Internal Medicine, Cardarelli Hospital, Campobasso, Italy; ^5^Cardiology Unit, San Camillo Hospital, Rome, Italy

**Keywords:** atrial fibrillation, DOACs, CHADS2, elderly, heart failure, risk index

## Abstract

**Background:** The aim of the study was to evaluate the efficacy and safety of direct oral anticoagulants (DOACs) in a subgroup of patients with atrial fibrillation (AF), CHADS_2_ score ≥3, advanced age, and heart failure (HF) coming from the main DOACs randomized clinical trials.

**Methods:** We searched MEDLINE, MEDLINE In-Process, and Other Non-Indexed Citations, EMBASE, PubMed, and the Cochrane Central Register of Controlled Trials. English-language articles published from 2002 to March 2019 dealing with DOACs for preventing thrombotic events in AF were considered. We did not conduct any statistical analyses, as indirect comparison between DOACs represents hypothesis generators.

**Results:** This systematic review was restricted to the subgroup of patients with CHADS_2_ score ≥3 (*n* = 31,203), elderly (*n* = 24,788), and with HF (*n* = 29,297) derived from the pivotal trials. Risk index (RI) was calculated. The RI for stroke/systemic embolism was similar in all of the patients treated with DOACs or warfarin. The lowest RI was in rivaroxaban patients (CHADS_2_ score ≥3: RI = 0.04; elderly: RI = 0.09; HF: RI = 0.05). The RIs for bleeding were higher in patients treated with dabigatran (CHADS_2_ score ≥3: RI_110_ = 0.23; elderly: RI_110_ = 0.22; HF: RI_110_ = 0.16; CHADS_2_score ≥3: RI_150_ = 0.30; elderly: RI_150_ = 0.24; HF: RI_150_ = 0.16). The bleeding RIs were higher with apixaban (CHADS_2_ score ≥3: RI = 0.23; elderly: RI = 0.25; HF: RI = 0.14) and dabigatran (CHADS_2_ score ≥3: RI = 0.28; elderly: RI = 0.21; HF: RI = 0.19).

**Conclusions:** The use of DOACs is a reasonable alternative to vitamin K antagonists in AF patients with CHADS_2_ score ≥3, advanced age, and HF. The RI constitutes a useful, additional tool to facilitate clinicians in choosing DOACs or warfarin in particular category of AF patients.

## Introduction

Atrial fibrillation (AF) is associated with high risk for stroke and systemic embolism. The prevention of these complications was carried out with long-term anticoagulant therapy. Until a few years ago, the therapeutic standard was dose-adjusted vitamin K antagonists (VKAs). Recently, four direct oral anticoagulants (DOACs) (dabigatran, rivaroxaban, apixaban, and edoxaban) extended the armamentarium of physicians in thromboprophylaxis of AF. They were approved following the results from their respective dose-adjusted phase III, warfarin controlled, randomized controlled trials (RCTs) (Connolly et al., 2009; Granger et al., 2011; Patel et al., 2011; Giugliano et al., 2013).

Given the comparable efficacy and safety profile of the four DOACs as compared to warfarin, the differences within the enrolled populations [such as percentages in patients with CHADS_2_ score ≥3, advanced age, and/or heart failure (HF)] should be taken into account in order to correctly tailor the therapy for the patients (Gage et al., 2001). The percentage of patients with CHADS_2_ score ≥3 within the four trials was 32.2% in the Randomized Evaluation of Long-Term Anticoagulant Therapy (RE-LY) study, 86.9% in the Rivaroxaban Once-daily oral direct factor Xa inhibition Compared with vitamin K antagonism for prevention of stroke and Embolism Trial in Atrial Fibrillation (ROCKET-AF) study, 30.2% in the Apixaban for Reduction in Stroke and Other Thromboembolic Events in Atrial Fibrillation (ARISTOTLE) study, and 53.1% in the Effective Anticoagulation with Factor Xa Next Generation in Atrial Fibrillation (ENGAGE-AF TIMI 48) study. The percentage of elderly patients was 40% in the RE-LY, 43.7% in the ROCKET-AF, 31.2% in the ARISTOTLE, and 40.1% in the ENGAGE-AF TIMI 48. Patients with HF were 32% in the RE-LY, 62.5% in the ROCKET-AF, 35.4% in the ARISTOTLE, and 57.9% in the ENGAGE-AF TIMI 48.

The aim of this systematic review was to evaluate the efficacy and safety of DOACs as compared to warfarin in a subgroup of patients with AF and CHADS_2_ score ≥3, advanced age, and HF.

We applied the risk index (RI) proposed by Uguccioni et al. (2018) to evaluate the efficacy and safety of DOACs and warfarin in patients at high thromboembolic risk who were enrolled in the pivotal studies.

## Methods

A comprehensive literature search was performedto identify RCTs reporting all-cause of stroke and systemic embolism and major bleeding in patients with AF, randomized to either VKA or DOAC, with CHADS_2_ score ≥3, age ≥75 years, and HF. We searched MEDLINE, MEDLINE In-Process, and Other Non-Indexed Citations. EMBASE, PubMed, and the Cochrane Central Register of Controlled Trials were searched through the Ovid interface to identify English-language clinical articles published from 2002 (first DOAC on the market) to March 2019 for all phase III RCT of patients receiving dabigatran, rivaroxaban, apixaban, or edoxaban versus warfarin for the prevention of thrombotic events in AF. Keywords were “atrial fibrillation,” “warfarin,” “oral thrombin inhibitor,” “oral factor Xa inhibitor,” “dabigatran,” “rivaroxaban,” “apixaban,” “edoxaban,” “CHADS_2_,” “Elderly,” and “Heart Failure.”

Regular alerts were also established. The electronic search strategy was complemented by a direct, manual review of the references.

Systematic reviews were eligible for inclusion if they included RCTs that evaluated stroke/SE and/or major bleeding, and evaluated DOACs and VKAs. Patient populations in eligible systematic reviews were required to include ≥90% of patients with nonvalvular AF (NVAF) or report results for NVAF populations separately. RCTs conducted to date evaluating DOACs have focused on patients with NVAF. Patients, intervention, comparator, outcomes, and study design (PICOS) criteria for inclusion and exclusion of NMAs are described in **Table 1** and **Supplementary Materials**.

**Table 1:** PICOS criteria for inclusion and exclusion of systematic review.

	Inclusion Criteria	Exclusion Criteria
Population	Patients with NVAF receiving any of the treatments below. All studies in the SLR must include ≥90% patients with NVAF. SLRs including studies with <90% patients with NVAF must report data separately for the NVAF studies	Not a population of interest (i.e., non-NVAF patients) Studies of patients receiving ablation, cardioversion, or left-atrial appendage closure
Intervention/comparator	DOACs (apixaban, dabigatran, rivaroxaban, edoxaban) and warfarin studies need to have compared 1 or more DOACs and/or warfarin	Studies not reporting outcomes for population of interest
Outcome	Clinical outcomes: • Stroke/systemic embolism • Major bleeding (ISTH or modified ISTH). • Patients with CHADS_2_ ≥3 • Elderly patients (age ≥ 75 years) • Patients with heart failure Doses included: Apixaban: 5 mg or 2.5 mg^a^ Rivaroxaban: 20 mg or 15 mg Dabigatran: 150 mg or 110 mg Edoxaban: 60 mg	SLRs/NMAs of observational studies, nonsystematic reviews, primary research trials, primary observational studies, case reports, case series, narrative reviews Letters to the editor, guidelines, meeting abstracts *In vitro* pharmacodynamic or pharmacokinetic studies only, animal studies, genetic studies only
Study design	SLR of randomized controlled trials	

PICOS, patients, intervention, comparator, outcomes, study design; DOAC, direct oral anticoagulant; ISTH, International Society on Thrombosis and Hemostasis; CHADS_2_, [congestive heart failure, hypertension, age ≥75 years, diabetes mellitus, stroke (double weight)]; NMA, network meta-analysis; NVAF, nonvalvular atrial fibrillation; SLR, systematic literature review.

^a^Any network meta-analysis comparison of apixaban 2.5 mg only with another DOAC was not included.

Search results were combined, and duplicates were removed. Studies were first screened on the basis of title and abstract; then, the full text was reviewed. Five reviewers (D.A., P.S., C.A., G.R., and G.C.) independently performed the revision, while discrepancies were solved by a consensus, contacting a further sixth author (M.M.C.).

**Figure 1:**
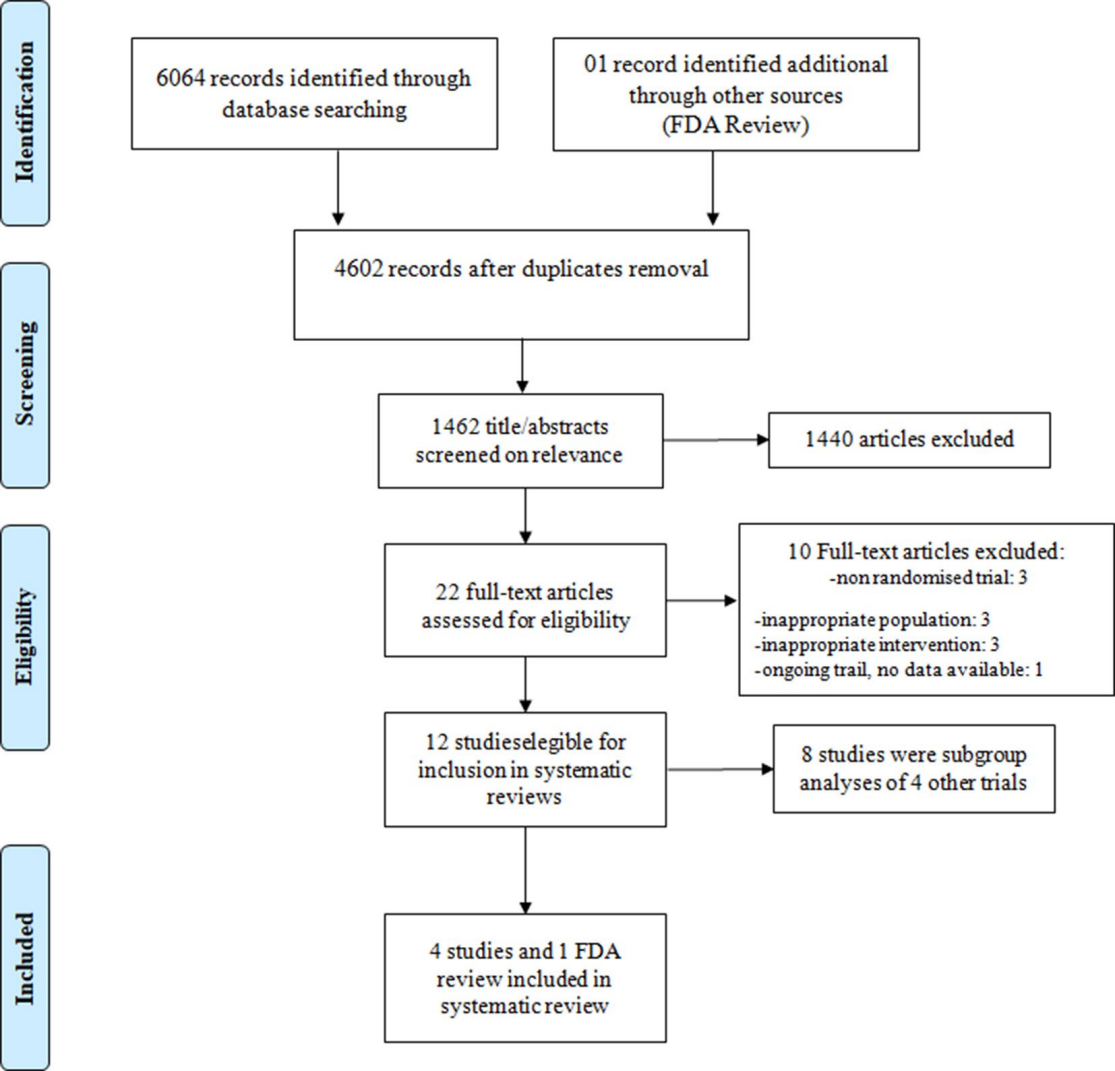
Preferred reporting items for systematic reviews and meta-analyses (PRISMA-P) flow diagram: search and selection process.

Data were derived from the four pivotal trials and one record of Food and Drug Administration (FDA) review (Connolly et al., 2009; FDA Medical Reviews, 2013; Patel et al., 2011; Granger et al., 2011; Apostolakis et al., 2013; Giugliano et al., 2013; Flaker et al., 2014; Goodman et al., 2014; Piccini et al., 2014; Held et al., 2015; De Caterina et al., 2016; Kato et al., 2016; Proietti et al., 2018) (**Figure 1**).

We computed the RI in relation to the efficacy (rate of stroke-systemic embolism/rate of patients with CHADS_2_ ≥3; rate of stroke–systemic embolism/rate of patients aged ≥75 years; rate of stroke–systemic embolism/rate of patients with HF) and safety (rate of major bleeding/rate of patients with CHADS_2_ ≥3; rate of major bleeding/rate of patients aged ≥75 years; rate of major bleeding/rate of patients with HF) of DOACs.

## Statistical Analysis

We did not conduct any statistical analyses because, in our opinion, the indirect comparison meta-analysis between DOACs represents hypothesis generators and cannot provide definitive answers.

On the other hand, the RI does not allow the comparisons of rates among nonhomogeneous studies.

## Main Results

We included 31,203 patients with AF and CHADS_2_ ≥3, 24,788 elderly patients, and 29,297 patients with AF and HF. All of them were enrolled into the main four randomized trials, comparing the incidence of stroke/systemic embolism and major bleeding of dabigatran (110 and 150 mg) BID, rivaroxaban 20 mg QD, apixaban 5 mg BID, and edoxabanhigh dose (60 mg) QD with warfarin.

**Figure 2:**
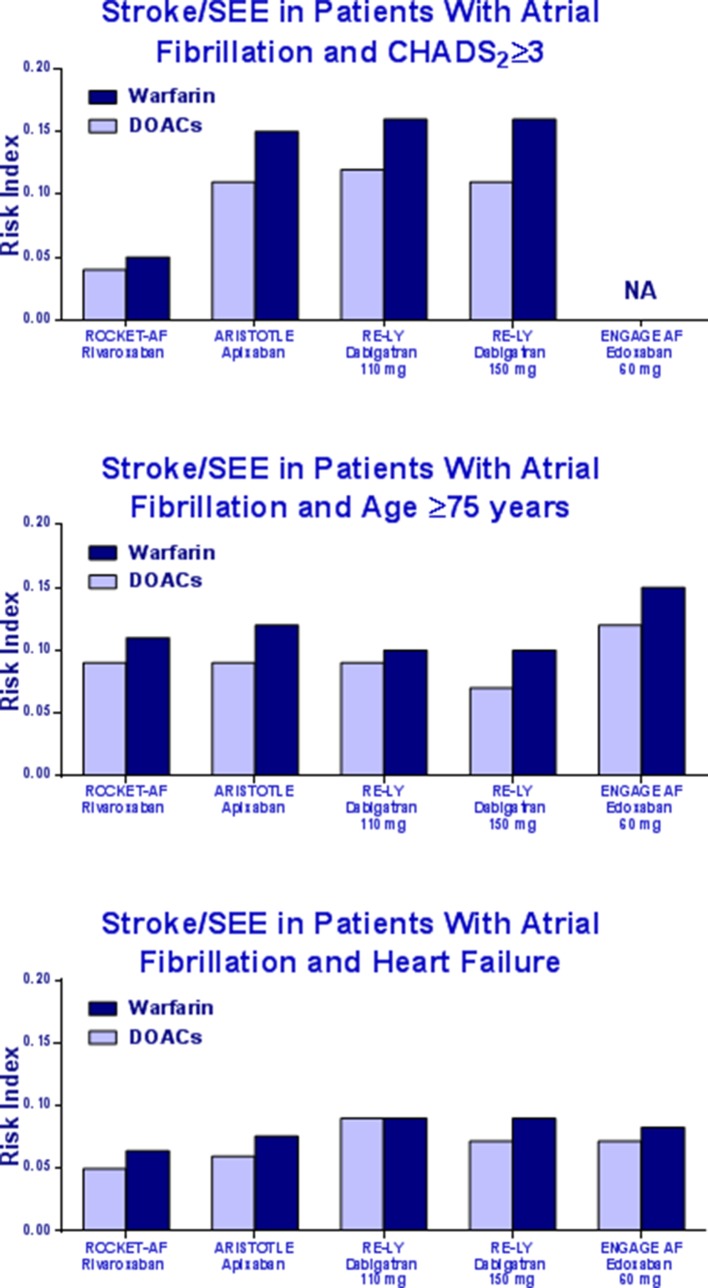
Risk Index of stroke/systemic embolism in patients with CHADS_2_ ≥3, elderly, or heart failure in the pivotal trials.

The RI of stroke/systemic embolism was similar between patients treated with DOACs and those who underwent warfarin treatment (**Figure 2**).

**Table 2** summarizes the results of our systematic review. The percentage of patients with CHADS_2_ ≥3 ranged from 30.2 to 87%. The highest frequency of CHADS_2_ ≥3 was in the group of patients treated with rivaroxaban (87%), whose RI value for major bleeding was lower as compared to other DOACs. In the RE-LY study, the rate of major bleeding with dabigatran was approximately five times higher than rivaroxaban. Bleeding data for the ENGAGE study according to CHADS_2_ ≥3 is not available.

**Table 2:** Stroke, systemic embolism, and major bleeding in patients with CHADS_2_ ≥3 in the pivotal trials.

Direct Oral Anticoagulants					
*RCTs*	*Pts on DOAC* *(N)*	*CHADS* *_2_* * ≥3%* *% (N)*	*Stroke o SEE* *CHADS* *_2_* * ≥3* *% (N)*	*MB* *CHADS* *_2_* *≥3%(N)*	*RI* *_efficacy_*	*RI* *_safety_*
Warfarin					
RCTs	Pts on WKA (N)	CHADS_2_≥3 % (N)	Stroke o SEE CHADS_2_ ≥3 % (N)	MB CHADS_2_ ≥3 %(N)	RI_efficacy_	RI_safety_	
ROCKET AF^2^	7,131	87% (6,205)	3.85% (239)	5.4% (337)	0.04	0.06
ARISTOTLE^3^	9,120	30.2% (2,758)	3.40% (94)	5.2% (143)	0.11	0.17
RE-LY_110_^1^	6,015	32.4% (1,951)	4.2% (82)	7.5% (147)	0.12	0.23
RE-LY_150_^1^	6,076	32.3% (1,965)	3.76% (74)	9.6% (188)	0.11	0.30
ENGAGE_HD_^4^	7,035	53.7% (3,784)	NA	NA	NA	NA
ROCKET AF^2^	7,133	86.8% (6,197)	4.35% (270)	5.4% (337)	0.05	0.06
ARISTOTLE^3^	9,081	30.2% (2,744)	4.81% (132)	6.9% (188)	0.15	0.23
RE-LY_110_ **_–_**_150_^1^	6,022	31.7% (1,914)	5.27% (101)	9.0% (172)	0.16	0.28
ENGAGE_HD_^4^	7,036	52.3% (3,685)	NA	NA	NA	NA

CHADS_2_, [congestive heart failure, hypertension, age ≥75 years, diabetes mellitus, stroke (double weight)]; RCTs, randomized controlled trials; DOAC, non-VKA antagonist drugs; N, number; Pts, patients; SEE, systemic embolism; MB, major bleeding; HD, higher dose; RI_efficacy_, Risk Index (rate of stroke–systemic embolism/rate of patients with CHADS_2_ ≥3; RI_safety_, Risk Index (rate of major bleeding/rate of patients with CHADS_2_ ≥3); NA, not available.

The elderly patients (age ≥75 years) enrolled in the four RCTs ranged from 31.1 to 43.8%. The highest frequency was in the ROCKET-AF trial. Bleeding RI was lower in patients treated with rivaroxaban as compared to other DOACs (**Table 3**).

**Table 3:** Stroke, systemic embolism, and major bleeding in elderly patients (age ≥75 years) in the pivotal trials.

Direct Oral Anticoagulants					
RCTs	Pts on DOAC (N)	≥75 years % (N)	Stroke or SEE ≥75 years % (N)	MB ≥75 years % (N)	RI_efficacy_	RI_safety_
Warfarin					
RCTs	Pts on WKA (N)	≥75 years % (N)	Stroke or SEE ≥75 years % (N)	MB ≥75 years % (N)	RI_efficacy_	RI_safety_
ROCKET AF^2^	7,131	43.8% (3,120)	4.00% (125)	7.15% (223)	0.09	0.16
ARISTOTLE^3^	9,120	31.2% (2,850)	2.77% (79)	5.3% (151)	0.09	0.17
RE-LY_110_^1^	6,015	39.1% (2,349)	3.7% (87)	8.7% (204)	0.09	0.22
RE-LY_150_^1^	6,076	40.6% (2,466)	2.79% (69)	9.97% (246)	0.07	0.24
ENGAGE_HD_^4^	7,035	40.5 (2,838)	5.00% (142)	7.68% (218)	0.12	0.19
ROCKET AF^2^	7,133	43.6% (3,109)	4.95% (154)	6.56% (204)	0.11	0.15
ARISTOTLE^3^	9,081	31.1% (2,828)	3.85% (109)	7.9% (224)	0.12	0.25
RE-LY_110–150_^1^	6,022	40.2% (2,423)	4.17% (101)	8.5% (206)	0.1	0.21
ENGAGE_HD_^4^	7,036	39.8 (2,805)	5.98.% (168)	9.30% (261)	0.15	0.23

RCTs, randomized controlled trials; DOAC, non-VKA antagonist drugs; N, number; Pts, patients; SEE, systemic embolism; MB, major bleeding; HD, higher-dose; RI_efficacy_, Risk Index (rate of stroke–systemic embolism/rate of patients aged ≥75 years); RI_safety_, Risk Index (rate of major bleeding/rate of patients aged ≥75 years).

The percentage of patients with HF ranged from 31.8 to 62.2% across the four pivotal studies. Patients treated with rivaroxaban showed a bleeding RI value lower than the RI values of apixaban, dabigatran, and edoxaban, respectively (**Table 4**).

**Table 4:** Stroke, systemic embolism, and major bleeding in patients with heart failure in the pivotal trials.

Direct Oral Anticoagulants												
RCTs	Pts on DOAC (N)	HF % (N)	Stroke o SEE HF % (N)	MB HF % (N)	RI_efficacy_	RI_safety_
Warfarin					
RCTs	Pts on WKA (N)	HF % (N)	Stroke o SEE HF % (N)	MB HF % (N)	RI_efficacy_	**RI** **_safety_**
ROCKET AF^2^	7,131	62% (4,467)	3.67% (164)	5.21% (233)	0.05	0.084
ARISTOTLE^3^	9,120	35.5% (3,235)	2.10% (68)	3.49% (227)	0.06	0.098
RE-LY_110_^1^	6,015	32.2% (1,937)	3.09% (60)	5.31% (103)	0.09	0.164
RE-LY_150_^1^	6,076	31.8% (1,934)	2.32% (45)	5.01% (97)	0.072	0.157
ENGAGE_HD_^4^	7,035	58.2 (4,097)	4.19% (172)	5.54% (227)	0.072	0.095
ROCKET AF^2^	7,133	62.2% (4,441)	4.0% (179)	5.24% (233)	0.064	0.084
ARISTOTLE^3^	9,081	35.4% (3,216)	2.7% (88)	4.85% (156)	0.076	0.14
RE-LY_110–150_^1^	6,022	31.9% (1,922)	3.06% (59)	6.24% (120)	0.09	0.19
ENGAGE_HD_^4^	7,036	57.5% (4,048)	4.8% (194)	7.0% (285)	0.083	0.12

RCTs, randomized controlled trials; DOAC, non-VKA antagonist drugs; N, number; Pts, patients; SEE, systemic embolism; HF, heart failure; MB, major bleeding; HD, higher dose; RI_efficacy_, Risk Index (rate of stroke–systemic embolism/rate of patients with heart failure); RI_safety_, Risk Index (rate of major bleeding/rate of patients with heart failure).

In the end, rivaroxaban showed a better safety RI as compared to other DOACs and similar to that of warfarin in patients with CHADS_2_ score ≥3. The RI in the ARISTOTLE, RE-LY, and ENGAGE is about three times higher than the RI derived from the ROCKET-AF trial. The same was in elderly and HF patients.

## Discussion

The choice of the correct antithrombotic/anticoagulant therapy in AF patients remains challenging, above all in higher risk patients such as those with CHADS_2_ score ≥3, older (age ≥75 years), and with HF.

The use of RI as proposed by Uguccioni et al. (2018) could minimize the heterogeneity of the patients enrolled in the RCTs and allow a better approach for choosing the correct anticoagulant in relation to the different characteristics of the patients. In particular, RI <1 indicates a favorable effect of the treatment. This is the first report dealing with risk of stroke/systemic embolism and major bleeding in patients with AF and CHADS_2_ ≥3, age ≥75, and HF assessed by RI.

In the present systematic review, all of the DOACs and warfarin seem to be efficient in preventing stroke and systemic embolism, showing lower rate of major bleeding. Indeed, the RI of each drug is <1, although some differences should be outlined.

Evidence shows different reproducibility rates of the data from DOACs clinical trials (both phase IV studies and “real-life” ones), above all in terms of safety. The incidence of frailty can largely account for the differences in performances. A meta-analysis from Ruff et al. (2014) involving the 71,683 patients with AF from the registration trials pointed out the significant reduction in stroke and systemic embolism incidence [relative risk (RR), 0.81; confidence interval (CI) 95%, 0.73–0.91; *p* < 0.0001] in DOACs patients as compared to warfarin, as well as all-causes mortality (RR, 0.90; CI 95%, 0.85–0.95; *p* = 0.0003), and intracranial hemorrhages (RR, 0.48; 95% CI, 0.39–0:59; *p* < 0.0001), despite the increase in gastrointestinal bleeding (RR, 1.25; 95% CI, 1.01–1.55; *p* = 0.04). In addition, reduced DOAC doses (dabigatran 110 mg BID or edoxaban 30 mg/day) showed similar results in terms of overall reduction of stroke and systemic embolism (RR, 1.03; 95% CI, 0.84–1.27; *p* = 0.74) and bleeding occurrence (RR, 0.65; 95% CI, 0:43–1:00; *p* = 0.05), despite the increase in ischemic stroke events (RR, 1.28; 95% CI, 1.02–1.60; *p* = 0.045).

Unfortunately, differences among studies had not been objectively evaluated. There are several methodological discrepancies (study design), different selection of the populations, and various definitions in outcomes among the four phase III RCTs (Connolly et al., 2009; Patel et al., 2011; Granger et al., 2011; Giugliano et al., 2013). Therefore, we tried to extrapolate the patients with high thromboembolic risk (CHADS_2_ score ≥3), advanced age (≥75 years), and HF in order to create a subset of uniform population.

The four registration trials differ according to the thromboembolic risk of enrolled populations. The highest rates of patients with CHADS_2_ score ≥3 were in ROCKET-AF (87%) and ENGAGE (52%) trials, while only one-third of ARISTOTLE and RE-LY patients had CHADS_2_ ≥3 (30 and 32%, respectively).

The incidence of major bleeding was similar in patients treated with rivaroxaban or warfarin in patients with CHADS_2_ score ≥3, while the incidence of major bleeding was higher in both dabigatran 110 mg, dabigatran 150 mg, and warfarin populations. This difference in the incidence of major bleeding appeared to be independent from the dose but rather linked to the patient’s risk profile, as well as factors that may influence pharmacokinetics and pharmacodynamics (comorbidities, advanced age, HF, hepatic or renal insufficiency). It is not possible to extrapolate data on major bleedings from the ENGAGE study as they were not available.

Indeed, a significant reduction in major bleeding in the ARISTOTLE study (apixaban 4.07%/year vs. warfarin 6.01%/year) and the ENGAGE study (edoxaban 2.75%/year vs. warfarin 3.43%/year) might be related to the large number of Asian patients (16%) in these trials (Gómez-Outes et al., 2016), as they have higher warfarin sensitivity; thus, the warfarin population could show the highest hemorrhagic events rates (Shen et al., 2007; Acanfora et al., 2018). Asian patients may require lower initiation and maintenance doses of warfarin.

Chronological age is often less important than the biological or physiological age of patients, i.e., health and physical conditions of the enrolled populations can influence results. Frailty can effectively influence the pharmacodynamicsof DOACs. It is mainly observed in >75-year-old patients, as they commonly show chronic diseases, comorbidities, functional decline, polypharmacy, and/or social/health problems.

On parallel, the prevalence of AF increases with age (Morillo et al., 2017), becoming the most frequent arrhythmia in >75-year-old patients (incidence rate, 10%) (Mitrousi et al., 2013). The risk for stroke also increases with age, independently from AF occurrence. Therefore, the elderly are at high risk of both stroke and AF, the association of these two conditions implementing the frailty condition. For this reason, age is included as a risk factor in most commonly used scores for the assessment of both thromboembolic and hemorrhagic risk in patients with AF (CHADS-VASc and HAS-BLED).

Furthermore, polypharmacy accounts for serious consequences due to drug-to-drug interactions. Oral anticoagulant therapy is the gold standard for the prevention of stroke in patients with AF, but it is largely underused (Lefebvre et al., 2016), even if the net clinical benefit in these patients has been already demonstrated (Alnsasra et al., 2019). In fact, oral anticoagulants are prescribed in 55% of the total population of high-risk AF patients, 35% in patients over 85 years old (Lefebvre et al., 2016). There is an inverse correlation between anticoagulants prescription and advanced age, which is considered as an independent predictor for nonprescription (Lefebvre et al., 2016). The reasons for the underutilization of anticoagulants in the elderly rely on the fear of potential bleedings. The incidence of VKA bleeding increases up to 5% per year in patients older than 75 years old (Patti et al., 2017). Nevertheless, the risk of bleeding did not increase in patients aged 80–89 years as compared to patients aged 70–79 years, while a 26% increase was observed in patients older than 90 years old (Patti et al., 2017).

Intracranial bleeding is 2.5 times more common in patients over 85 years of age: they account for 90% of deaths or severe disability. The highest bleeding risk with AVKs led to underutilization of oral anticoagulants in the elderly. The main advantage for using DOACs over VKA was the reduction in intracranial bleeding rate (Connolly et al., 2009; Patel et al., 2011; Granger et al., 2011; Giugliano et al., 2013).

The increased risk of falling influences the reduction in prescribing anticoagulants. In oral anticoagulated patients, subarachnoid hemorrhages related to falls are rare in elderly subjects with AF and CHADS-VASc >5. It has been calculated that elderly patient should fall more than 300 times a year before overcoming the clinical benefit of oral anticoagulation (Man-Son-Hing et al., 1999).

Recently, (Chao et al., 2018) showed that oral anticoagulant therapy was associated with a lower risk for ischemic stroke, lower risk for intracranial hemorrhage, and a clear net clinical benefit in patients with AF and age ≥90 years (Chao et al., 2018). Therefore, the authors emphasized the use of DOACs for thromboprophylaxis in very elderly patients (Chao et al., 2018). Coleman et al. demonstrated that the use of rivaroxaban, but not apixaban or dabigatran, was associated with a reduction in stroke and systemic embolism as compared to warfarin in frail elderly patients (Coleman et al., 2017). In addition, elderly frail patients with venous thromboembolism treated with rivaroxaban showed reduced thromboembolic relapses and had a better impact on bleeding than warfarin (Martinez et al., 2018).

Indeed, the incidence of HF failure increases with age is related to the increase in AF incidence, and the combination of HF and AF promotes the declines in patients’ prognosis (Sartipy et al., 2017). The need for anticoagulation is the mainstay in the general optimization of the therapy of these patients. Nevertheless, registries provide data about underused administration of DOACs in AF and HF patients (Savarese et al., 2018). The reasons for misleading adoption of these drugs are medieval, to some extent: the fear for bleedings is the main conditioner to DOACs underuse, as outlined by Savarese et al. (2018). Instead, Savarese et al. (2016) observed reduced incidence in intracranial and any bleeding in patients with HF and AF, even compared to patients without HF. Furthermore, DOACs are even able to significantly reduce efficacy and safety endpoints in HF patients (Savarese et al., 2016), this providing solid data about the need for the use of DOACs in patients with HF.

Among AF patients with HF, Xiong et al. (2015) shows that single-/high-dose DOAC regimens have significantly better efficacy and safety profiles as compared to warfarin. Low-dose regimens had similar efficacy and safety as compared to warfarin. Finally, DOACs were similarly effective and, to some extent, safer (reduced incidence of intracranial hemorrhages) in AF patients with HF as compared to those without HF (Xiong et al., 2015).

## Conclusion

Our data showed that, if the patients of the four phase III RCTs with NOACs were stratified according to CHADS_2_ score ≥3, advanced age, or HF, rivaroxaban had the lowest RI of major bleeding.

The use of DOACs is a reasonable alternative to vitamin K antagonists in the management of patients with AF and CHADS_2_ score ≥3, advanced age, and HF.

The risk index constitutes a useful additional tool to facilitate the clinicians in choosing DOACs or warfarin in particular category of AF patients.

## Author Contributions

DA, MC, CA, GR, GC, PS conceived and designed the study, analysed and interpreted the statistical data, drafted the article စ and critically reviewed its intellectual content, and finally approved the version to be submitted for publication. MU improved the work in relation to reviewers' comments, analysed and interpreted the new statistical data, drafted the revised version of the article and critically reviewed its intellectual content, and finally approved the version to be submitted for publication.

## Conflict of Interest Statement

The authors declare that the research was conducted in the absence of any commercial or financial relationships that could be construed as a potential conflict of interest.
