# Supplementary material for: Efficacy and Safety of Direct Oral Anticoagulants in Patients With Atrial Fibrillation and High Thromboembolic Risk. A Systematic Review
Source: Front Pharmacol. 2019 Sep 19;10:1048. doi: 10.3389/fphar.2019.01048 (PMC6761253; doi:10.3389/fphar.2019.01048)
Supplement: Supplementary file 1 [file DataSheet_1.docx]

# Details of the search strategy

**S1: Search strategy according to PRISMA-P**

| **OVERVIEW** | |
| --- | --- |
|  |  |
| Interface | Ovid |
|  |  |
|  | EBM Reviews - Cochrane Central Register of Controlled Trials |
|  | Ovid Embase |
| Databases | Ovid MEDLINE |
|  | Ovid MEDLINE Daily |
|  |  |
|  | Ovid MEDLINE In-Process & Other Non-Indexed Citations |
|  |  |
|  | *Note: Duplicates between databases were removed in Ovid.* |
|  | PubMed |
|  |  |
| Date of | 01-9-2018 |
| search |  |
|  |  |
| Alerts | Monthly search updates began 01-9-2018 and ran until project completion. |
| Study types | Randomized Controlled Trials |
|  | English language |
| Limits | Publication years 2002-present |
|  | Conference abstracts omitted |
|  |  |

## S2: Medline

Ovid MEDLINE(R) In-Process & Other Non-Indexed Citations, Ovid MEDLINE(R) Daily, Ovid MEDLINE(R) and Ovid OLDMEDLINE(R) 2002 to Present.

| **ID** | **PICOS** | **Category** | **Search terms** |
| --- | --- | --- | --- |
| #1 | P | Indication (VTEx) | venous thromboembolism/ |
| #2 |  |  | deep vein thrombosis/ |
| #3 |  |  | ((vein or venous) adj thromb$).ti,ab. |
| #4 |  |  | ((pulmonary or lung) adj embol$).ti,ab. |
| #5 |  |  | (dvt or vte or dvts or vtes).ti,ab. |
| #6 |  |  | or/1-5 |
| #7 |  | Indication (SPAF) | (spaf or nvaf).ti,ab. |
| #8 |  |  | Atrial Fibrillation/ or Atrial Flutter/ |
| #9 |  |  | ((Atrial or atrium or auricular) adj2 fibrillat$).ti,ab. |
| #10 |  |  | (AF or A-fib or a fib).mp. |
| #11 |  |  | or/7-10 |
| #12 |  | Indication (VTEp OS) | exp arthroplasty, replacement, knee/ |
| #13 |  |  | (knee adj (replacement or arthroplasty or prosthesis or joint)).mp. |
| #14 |  |  | TKR.mp. |
| #15 |  |  | exp arthroplasty, replacement, hip/ |
| #16 |  |  | (hip adj (replacement or arthroplasty or prosthesis or fracture)).mp. |
| #17 |  |  | THR.mp. |
| #18 |  |  | or/12-17 |
| #19 | Hits of P | 6 or 11 or 18 | 270,892 |
| #20 | I and C | Interventions and Comparators | antithrombins/ |
| #21 |  |  | noac$.mp. |
| #22 |  |  | new oral anticoagulant$.mp. |
| #23 |  |  | doac$.mp. |
| #24 |  |  | direct oral anticoagulant$.mp. |
| #25 |  |  | New orally active anticoagulant$.mp. |
| #26 |  |  | Novel oral anticoagulant$.mp. |
| #27 |  |  | 21 or 22 or 23 or 24 or 25 or 26 |
| #28 |  |  | IIa inhibitor$.mp. |
| #29 |  |  | direct thrombin inhibitor$.mp. |
| #30 |  |  | 28 or 29 |
| #31 |  |  | Factor Xa Inhibitor/ |
| #32 |  |  | Xa inhibitor$.mp. |
| #33 |  |  | fxa inhibitor$.mp. |
| #34 |  |  | factor 10a inhibitor$.mp. |
| #35 |  |  | 31 or 32 or 33 or 34 |
| #36 |  |  | (dabigatran or BIBR$1048 or pradax* or prazax*).mp. |
| #37 |  |  | (edoxaban or DU$176b or lixiana).mp. |
| #38 |  |  | (apixaban or BMS$562247 or eliquis).mp. |
| #39 |  |  | (rivaroxaban or BAY 59$7939 or xarelto).mp. |
| #40 |  |  | 36 or 37 or 38 or 39 |
| #41 | Hits of I and C | 20 or 27 or 30 or 35 or 40 |  |
| #42 | S | Observational studies | Epidemiologic studies/ |
| #43 |  |  | exp case control studies/ |
| #44 |  |  | exp cohort studies/ |
| #45 |  |  | Case control.tw. |
| #46 |  |  | (cohort adj (study or studies)).tw. |
| #47 |  |  | Cohort analy$.tw. |
| #48 |  |  | (Follow up adj (study or studies)).tw. |
| #49 |  |  | (observational adj (study or studies)).tw. |
| #50 |  |  | Longitudinal.tw. |
| #51 |  |  | Retrospective.tw. |
| #52 |  |  | Cross sectional.tw. |
| #53 |  |  | Cross-sectional studies/ |
| #54 |  |  | (cohort$1 or cross section$ or crosssection$ or (real adj1 (world or life))).tw. |
| #55 |  |  | (claim$ adj1 (data or analys*)).tw. |
| #56 |  |  | Registries/ |
| #57 |  |  | (database$ or registry or registries or effectiveness or prospective stud$).tw. |
| #58 |  |  | or/42-57 |
| #59 |  |  | Randomized controlled trial/ or Randomization/ |
| #60 |  |  | Comment.pt. |
| #61 |  |  | Letter.pt. |
| #62 |  |  | Editorial.pt. |
| #63 |  |  | Case reports.pt. |
| #64 |  |  | 59 or 60 or 61 or 62 or 63 |
| #65 | Hits of S | 58 not 64 |  |
| #66 | Hits of P and I and S | 19 and 41 and 65 |  |
| #67 | Limits | Humans | animals/ |
| #68 |  | 66 not 67 |  |
| #75 |  |  | remove duplicates from 68 |

## S3: Embase

Embase Classic and Embase 1947 to 2015 Week 21 using Ovid plateform

| **ID** | **PICOS** | **Category** | **Search terms** |
| --- | --- | --- | --- |
| #1 | P | Indication (VTEx) | venous thromboembolism/ |
| #2 |  |  | deep vein thrombosis/ |
| #3 |  |  | ((vein or venous) adj thromb$).ti,ab. |
| #4 |  |  | ((pulmonary or lung) adj embol$).ti,ab. |
| #5 |  |  | (dvt or vte or dvts or vtes).ti,ab. |
| #6 |  |  | or/1-5 |
| #7 |  | Indication (SPAF) | (spaf or nvaf).ti,ab. |
| #8 |  |  | Atrial Fibrillation/ or Atrial Flutter/ |
| #9 |  |  | ((Atrial or atrium or auricular) adj2 fibrillat$).ti,ab. |
| #10 |  |  | (AF or A-fib or a fib).mp. |
| #11 |  |  | or/7-10 |
| #12 |  | Indication (VTEp OS) | exp arthroplasty, replacement, knee/ |
| #13 |  |  | (knee adj (replacement or arthroplasty or prosthesis or joint)).mp. |
| #14 |  |  | TKR.mp. |
| #15 |  |  | exp arthroplasty, replacement, hip/ |
| #16 |  |  | (hip adj (replacement or arthroplasty or prosthesis or fracture)).mp. |
| #17 |  |  | THR.mp. |
| #18 |  |  | or/12-17 |
| #19 | Hits of P | 6 or 11 or 18 |  |
| #20 | I and C | Interventions and Comparators | antithrombins/ |
| #21 |  |  | noac$.mp. |
| #22 |  |  | new oral anticoagulant$.mp. |
| #23 |  |  | doac$.mp. |
| #24 |  |  | direct oral anticoagulant$.mp. |
| #25 |  |  | New orally active anticoagulant$.mp. |
| #26 |  |  | Novel oral anticoagulant$.mp. |
| #27 |  |  | 21 or 22 or 23 or 24 or 25 or 26 |
| #28 |  |  | IIa inhibitor$.mp. |
| #29 |  |  | direct thrombin inhibitor$.mp. |
| #30 |  |  | 28 or 29 |
| #31 |  |  | Factor Xa Inhibitor/ |
| #32 |  |  | Xa inhibitor$.mp. |
| #33 |  |  | fxa inhibitor$.mp. |
| #34 |  |  | factor 10a inhibitor$.mp. |
| #35 |  |  | 31 or 32 or 33 or 34 |
| #36 |  |  | (dabigatran or BIBR$1048 or pradax* or prazax*).mp. |
| #37 |  |  | (edoxaban or DU$176b or lixiana).mp. |
| #38 |  |  | (apixaban or BMS$562247 or eliquis).mp. |
| #39 |  |  | (rivaroxaban or BAY 59$7939 or xarelto).mp. |
| #40 |  |  | 36 or 37 or 38 or 39 |
| #41 | Hits of I and C | 20 or 27 or 30 or 35 or 40 |  |
| #42 | S | Observational studies | Epidemiologic studies/ |
| #43 |  |  | exp case control studies/ |
| #44 |  |  | exp cohort studies/ |
| #45 |  |  | Case control.tw. |
| #46 |  |  | (cohort adj (study or studies)).tw. |
| #47 |  |  | Cohort analy$.tw. |
| #48 |  |  | (Follow up adj (study or studies)).tw. |
| #49 |  |  | (observational adj (study or studies)).tw. |
| #50 |  |  | Longitudinal.tw. |
| #51 |  |  | Retrospective.tw. |
| #52 |  |  | Cross sectional.tw. |
| #53 |  |  | Cross-sectional studies/ |
| #54 |  |  | (cohort$1 or cross section$ or crosssection$ or (real adj1 (world or life))).tw. |
| #55 |  |  | (claim$ adj1 (data or analys*)).tw. |
| #56 |  |  | Registries/ |
| #57 |  |  | (database$ or registry or registries or effectiveness or prospective stud$).tw. |
| #58 |  |  | or/42-57 |
| #59 |  |  | Randomized controlled trial/ or Randomization/ |
| #60 |  |  | Comment.pt. |
| #61 |  |  | Letter.pt. |
| #62 |  |  | Editorial.pt. |
| #63 |  |  | Case reports.pt. |
| #64 |  |  | 59 or 60 or 61 or 62 or 63 |
| #65 | Hits of S | 58 not 64 |  |
| #66 | Hits of P and I and S | 19 and 41 and 65 |  |
| #67 | Limits | Humans | animals/ |
| #68 |  | 66 not 67 |  |
| #75 |  |  | remove duplicates from 68 |

## S4: Cochrane

Cochrane Database of Systematic Reviews, Database of Abstracts of Reviews of Effect, Cochrane Central Register of Controlled Trials, Health Technology Assessment Database and NHS Economic Evaluation Database

| **ID** | **PICOS** | **Category** | **Search terms** |
| --- | --- | --- | --- |
| #1 | P | Indication (VTEx) | [mh "Venous Thromboembolism"] |
| #2 |  |  | [mh "deep vein thrombosis"] |
| #3 |  |  | ((vein or venous) near thromb*) |
| #4 |  |  | ((pulmonary or lung) near embol*) |
| #5 |  |  | (dvt or vte or dvts or vtes) |
| #6 |  |  | #1 or #2 or #3 or #4 or #5 |
| #7 |  | Indication (SPAF) | (spaf or nvaf):ti,ab |
| #8 |  |  | [mh "Atrial Fibrillation"] |
| #9 |  |  | [mh "Atrial Flutter"] |
| #10 |  |  | ((Atrial or atrium or auricular) near/2 fibrillat*) |
| #11 |  |  | (AF or A-fib or a fib) |
| #12 |  |  | #7 or #8 or #9 or #10 or #11 |
| #13 |  | Indication (VTEp OS) | [mh "Arthroplasty, Replacement, Knee"] |
| #14 |  |  | (knee near (replacement or arthroplasty or prosthesis or joint)) |
| #15 |  |  | (TKR) |
| #16 |  |  | [mh "Arthroplasty, Replacement, Hip"] |
| #17 |  |  | (hip near (replacement or arthroplasty or prosthesis or fracture)) |
| #18 |  |  | (THR) |
| #19 |  |  | #13 or #14 or #15 or #16 or #17 or #18 |
| #20 | Hits of P | #6 or #12 or #19 |  |
| #21 | I and C | Interventions and Comparators | [mh antithrombins] |
| #22 |  |  | (noac*) |
| #23 |  |  | (new oral anticoagulant*) |
| #24 |  |  | (doac*) |
| #25 |  |  | (direct oral anticoagulant*) |
| #26 |  |  | (New orally active anticoagulant*) |
| #27 |  |  | (Novel oral anticoagulant*) |
| #28 |  |  | #22 or #23 or #24 or #25 or #26 or #27 |
| #29 |  |  | (IIa inhibitor*) |
| #30 |  |  | (direct thrombin inhibitor*) |
| #31 |  |  | #29 or #30 |
| #32 |  |  | [mh "Factor Xa Inhibitors"] |
| #33 |  |  | (Xa inhibitor*) |
| #34 |  |  | (fxa inhibitor*) |
| #35 |  |  | (factor 10a inhibitor*) |
| #36 |  |  | #32 or #33 or #34 or #35 |
| #37 |  |  | (dabigatran or BIBR$1048 or pradax* or prazax*) |
| #38 |  |  | (edoxaban or DU$176b or lixiana) |
| #39 |  |  | (apixaban or BMS$562247 or eliquis) |
| #40 |  |  | (rivaroxaban or BAY 59$7939 or xarelto) |
| #41 |  |  | #37 or #38 or #39 or #40 |
| #42 | Hits of I and C | #21 or #28 or #31 or #36 or #41 |  |
| #43 | S | Observational studies | [mh "Epidemiologic studies"] |
| #44 |  |  | [mh "case control studies"] |
| #45 |  |  | [mh "cohort studies"] |
| #46 |  |  | (Case control) |
| #47 |  |  | (cohort near (study or studies)) |
| #48 |  |  | (Cohort analy*) |
| #49 |  |  | (Follow up near (study or studies)) |
| #50 |  |  | (observational near (study or studies)) |
| #51 |  |  | (Longitudinal) |
| #52 |  |  | (Retrospective) |
| #53 |  |  | (Cross sectional) |
| #54 |  |  | [mh "Cross-sectional studies"] |
| #55 |  |  | (cohort* or cross section* or crosssection* or (real near/1 (world or life))) |
| #56 |  |  | (claim* near/1 (data or analys*)) |
| #57 |  |  | [mh Registries] |
| #58 |  |  | (database* or registry or registries or effectiveness or prospective stud*) |
| #59 |  |  | #43 or #44 or #45 or #46 or #47 or #48 or #49 or #50 or #51 or #52 or #53 or #54 or #55 or #56 or #57 or #58 |
| #60 |  |  | [mh "Randomized controlled trial"] |
| #61 |  |  | [mh Randomization] |
| #62 |  |  | (Comment):pt |
| #63 |  |  | (Letter):pt |
| #64 |  |  | (Editorial):pt |
| #65 |  |  | (Case reports):pt |
| #66 |  |  | #60 or #61 or #62 or #63 or #64 or #65 |
| #67 | Hits of S | #59 not #66 |  |
| #68 | Hits of P and I and S | #20 and #42 and #67 |  |
| #69 | Limits | Humans | [mh Animals] |
| #70 |  | #68 not #69 |  |

S4: Trial Registers

| *Trial Registers (date: 01-09-2018)* | |
| --- | --- |
| *Source* | *Search Strategy* |
| https://www.clinicaltrialsregister.eu/ | (Dabigatran OR Pradax OR Pradaxa OR apixaban OR Eliquis OR rivaroxaban OR Xarelto) AND (warfarin OR Coumadin OR acenocoumarol OR Sintrom OR Sinthrome) AND Atrial Fibrillation |
| ^https://clinicaltrials.gov/^ | (Dabigatran OR Pradax OR Pradaxa OR apixaban OR Eliquis OR rivaroxaban OR Xarelto) AND (warfarin OR Coumadin OR acenocoumarol OR Sintrom OR Sinthrome) AND Atrial Fibrillation |

S4: grey Literature

| *Grey Literature (date: 01-09-2018)* | |
| --- | --- |
| *Source* | *Search Strategy* |
| <http://www.opengrey.eu/search/> | (Dabigatran OR Pradax OR Pradaxa OR apixaban OR EliquisOR rivaroxaban OR Xarelto) AND (warfarin OR Coumadin ORacenocoumarol OR Sintrom OR Sinthrome) AND Atrial Fibrillation |
| <http://www.greylit.org/> | (Dabigatran OR Pradax OR Pradaxa OR apixaban OR EliquisOR rivaroxaban OR Xarelto) AND (warfarin OR Coumadin ORacenocoumarol OR Sintrom OR Sinthrome) AND Atrial Fibrillation |

**S5 (a): Risk of Bias Assessment**

| *Publication:* Dabigatran versus Warfarin in Patients with Atrial Fibrillation. N Engl J Med2009;361:1139-51.  *Authors:* Connolly SJ, Ezekowitz MD, Wallentin L. | | | | | | | | | | | | |
| --- | --- | --- | --- | --- | --- | --- | --- | --- | --- | --- | --- | --- |
| **Domain** | **Support for Judgement** | **Review Author’s Judgement** | | | | | | | | | | |
| **Selection Bias** | | | | | | | | | | | | |
| *Random*  *Sequence*  *Generation* | Insufficient information in article or rationale and design publication | High Risk | Unclear | | | | | Low Risk | | | | |
| *Allocation*  *Concealment* | Central interactive automated telephone system | High Risk | Unclear | | | | | Low Risk | | | | |
| **Performance Bias** | | | | | | | | | | | | |
| *Blindings of*  *partecipants*  *And personnel* | Incomplete blinding:   - Blinded to Dabigatran dose - Unblinded with respect to Dabi-gatran or warfarin assignment   All investigators, members of coordinating center, the operation committee, the stering committee, the event adjudication committee, and the sponsor remain blinded to treatment level analyses of efficacy and safety. Only the data and safety monitoring board (DSMB) and the DSMB-statistician have access to the randomization code and by-treatment event rates | High Risk | | Unclear | | | | | | | | Low Risk |
| **Detection Bias** | | | | | | | | | | | | |
| *Blinding of*  *Outcome*  *assestment* | Adjudication of endpoints is blinded to drug assignment; by a blinded adjudication committee. | High Risk | | | Unclear | | | | | | Low Risk | |
| **Attrition Bias** | | | | | | | | | | | | |
| *Incomplete*  *Outcome*  *data* | All analyses based on intention-to-treatprinciple. 20 patients (out of 18.113) lost tofollow-up. | High Risk | | | | Unclear | | | | Low  Risk | | |
| **Incomplete Bias** | | | | | | | | | | | | |
| *Selective*  *reporting* | Study protocol available and all pre-specified outcomes are reported.  The unexpectedly different rates of myocardial infarction and gastrointestinal bleeding among the three treatment groups support an absence of bias.  To detect possible unreported events, symptom questionnaires were regularly administered to patients, and adverse-event and hospitalization reports were scrutinized for unreported primary or secondary outcomes | High Risk | | | | | Unclear | | Low Risk | | | |

**S5 (b): Risk of Bias Assessment**

| *Publication: FDA MEDICAL REVIEWS. PRADAXA (dabigatran etexilate-mesylate) Capsules Company:Boehringer Ingelheim Pharmaceuticals, Inc. Application No.: 022512 Approval Date: 10/19/2010center for drug evaluation and research application number: 22-512 medical review(s).*[*Authors: Nhi Beasley, Aliza Thompson.*https://www.accessdata.fda.gov/drugsatfda_docs/nda/2010/022512Orig1s000MedR.pdf](https://www.accessdata.fda.gov/drugsatfda_docs/nda/2010/022512Orig1s000MedR.pdf) | | | | |
| --- | --- | --- | --- | --- |
| **Domain** | **Support for Judgement** | | **Review Author’s Judgement** | |
| **Selection Bias** | | | | |
| *Random*  *Sequence*  *Generation* | Not applicable, see section S5 (a) | High Risk | Unclear | Low Risk |
| *Allocation*  *Concealment* | Not applicable, see section S5 (a) | High Risk | Unclear | Low Risk |
| **Performance Bias** | | | | |
| *Blindings of*  *partecipants*  *And personnel* | Not applicable, see section S5 (a) | High Risk | Unclear | Low Risk |
| **Detection Bias** | | | | |
| *Blinding of*  *Outcome*  *assestment* | Not applicable, see section S5 (a) | High Risk | Unclear | Low Risk |
| **Attrition Bias** | | | | |
| *Incomplete*  *Outcome*  *data* | Not applicable, see section S5 (a) | High Risk | Unclear | Low Risk |
| **Incomplete Bias** | | | | |
| *Selective*  *reporting* | Not applicable, see section S5 (a) | High Risk | Unclear | Low Risk |

**S5 (c): Risk of Bias Assessment**

| *Publication:* Rivaroxaban versus Warfarin in Nonvalvular Atrial Fibrillation. N Engl J Med2011;365:883-91.*Authors:* Patel MR, Mahaffey KW, Califf RM. | | | | | | | | | | | |
| --- | --- | --- | --- | --- | --- | --- | --- | --- | --- | --- | --- |
| **Domain** | **Support for Judgement** | **Review Author’s Judgement** | | | | | | | | | |
| **Selection Bias** | | | | | | | | | | | |
| *Random*  *Sequence*  *Generation* | Insufficient information in article or rationale and design publication. | High Risk | Unclear | | | | | | | | Low Risk |
| *Allocation*  *Concealment* | Central 24-hour, computerized, automated voice-response system. | High Risk | Unclear | | | | | | | | Low Risk |
| **Performance Bias** | | | | | | | | | | | |
| *Blindings of*  *partecipants*  *And personnel* | Double-blind, double-dummy. A point-of-care device was used to either generate real INR values or sham values. Sham INR results were generated by means of a validated algorithm reflecting the distribution of values in warfarin-treated patients with characteristics similar to those in the study population. | High Risk | | Unclear | | | | | | | Low Risk |
| **Detection Bias** | | | | | | | | | | | |
| *Blinding of*  *Outcome*  *assestment* | An independent clinical end-point committee applied protocol definitions to adjudicate all suspected cases of stroke, systemic embolism, myocardial infarction, death and bleeding events that contributed to the prespecified end points. | High Risk | | | Unclear | | | | | Low Risk | |
| **Attrition Bias** | | | | | | | | | | | |
| *Incomplete*  *Outcome*  *data* | The primary efficacy analysis (noninferiority) will be undertaken in the per-protocol population, which comprises all randomized patients who have received study drug, except those who have major protocol violations before a primary end point event. The analysis population consists of randomized subjects who have taken at least one dose of study drug. Testing for noninferiority and superiority was also performed in the intention-to-treat population, which included all patients who underwent randomization and were followed for events during treatment or after premature discontinuation 32 patients lost to follow up (18 in rivaroxaban group, 14 in warfarin group). The proportions of patients who permanently stopped their assigned therapy before an end-point event and before the termination date were 23.7% in the rivaroxaban group and 22.2% in the warfarin group. Because of violations in GCP guidelines at one site that made the data unreliable, 93 patients (50 in the rivaroxaban group and 43 in the warfarin group) were excluded from all efficacy analyses before unblinding. | High Risk | | | | Unclear | | | Low Risk | | |
| **Incomplete Bias** | | | | | | | | | | | |
| *Selective*  *reporting* | Study protocol available and all pre-specified outcomes are reported. When randomized, all patients will be observed for the duration of the study to ascertain clinical events. Patients will be seen at 1, 2, and 4 weeks, and every month thereafter for detection of primary efficacy end point events, as well as TIA, MI, bleeding complications, procedures, and vital status evaluation. A standardized questionnaire and examination will be used to screen for stroke symptoms and clinical events that will prompt further evaluation. | High Risk | | | | | Unclear | Low Risk | | | |

**S5 (d): Risk of Bias Assessment**

| *Publication:* Apixaban versus Warfarin in Patients with Atrial Fibrillation. N Engl J Med 2011;365:981-92.*Authors:* Granger CB, Alexander JH, Wallentin L. | | | | | |
| --- | --- | --- | --- | --- | --- |
| **Domain** | **Support for Judgement** | **Review Author’s Judgement** | | | |
| **Selection Bias** | | | | | |
| *Random*  *Sequence*  *Generation* | Insufficient information in article or rationale and design publication. | High Risk | | Unclear | Low Risk |
| *Allocation*  *Concealment* | Insufficient information in article or rationale and and design publication. | High Risk | Unclear | | Low Risk |
| **Performance Bias** | | | | | |
| *Blindings of*  *partecipants*  *And personnel* | Double-blind, double-dummy. Blinded treatment is maintained through the use of sham international normalized ratios in patients receiving apixaban. | High Risk | Unclear | | Low Risk |
| **Detection Bias** | | | | | |
| *Blinding of*  *Outcome*  *assestment* | An independent, blinded, clinical events committee (CEC)adjudicates all suspected hemorrhagic and non- hemorrhagicstrokes, TIAs, systemic emboli, major and clinically relevantnon- major bleeding, myocardial infarction, and cause ofdeath. Using prespecified event definitions and agreed uponevent adjudication criteria, the CEC adjudicates suspectedevents based on the preponderance of the evidence and theclinical knowledge and experience of the physicianreviewers. | High Risk | Unclear | | Low Risk |
| **Attrition Bias** | | | | | |
| *Incomplete*  *Outcome*  *data* | The primary and secondary efficacy analyses included all patients who underwent randomization (intention-to-treat population) and included all events from the time of randomization until the cutoff date for efficacy outcomes. The analyses of bleeding events included all patients who received at least one dose of a study drug and included all events from the time the first dose of a study drug was received until 2 days after the last dose was received. In a modified intention-to-treat sensitivity analysis, we analyzed bleed- ing events that occurred in patientswho received at least one dose of a study drug andincluded all events from the time of randomizationuntil January 30, 2011.Data on vital status at the end of the trial were missing for 380 patients (2.1%). The absence of data on vital statuswas due to withdrawal of consent in the case of 92 patientsin the apixaban group (1.0%) and 107 patients in thewarfarin group (1.2%) and was due to loss to follow-up inthe case of 35 patients in the apixaban group (0.4%) and34 in the warfarin group (0.4%) | High Risk | Unclear | | Low Risk |
| **Incomplete Bias** | | | | | |
| *Selective*  *reporting* | Study protocol available and all pre-specifiedoutcomes are reported.  Visits every 3 months included an assessment of clinicaloutcomes and adverse events. For each patient who waslost to follow-up or who withdrew consent, attempts weremade to determine vital status at the end of the trial. | High Risk | | Unclear | Low Risk |

**S5 (e): Risk of Bias Assessment**

| *Publication:* Edoxaban versus Warfarin in Patients with Atrial Fibrillation. *Authors:* Giugliano RP, Ruff CT, Braunwald EMPH | | | | | | |
| --- | --- | --- | --- | --- | --- | --- |
| **Domain** | **Support for Judgement** | **Review Author’s Judgement** | | | | |
| **Selection Bias** | | | | | | |
| *Random*  *Sequence*  *Generation* | Insufficient information in article or rationale and designpublication. | High Risk | Unclear | | | Low Risk |
| *Allocation*  *Concealment* | All subjects are randomized through an interactive voice/Webresponse system. | High Risk | Unclear | | | Low Risk |
| **Performance Bias** | | | | | | |
| *Blindings of*  *partecipants*  *And personnel* | Double-blind, double-dummy.  Blinded treatment is maintained through the use of shaminternational normalized ratios in patients receiving edoxaban. | High Risk | | Unclear | Low Risk | |
| **Detection Bias** | | | | | | |
| *Blinding of*  *Outcome*  *assestment* | Cause of death, stroke, SEE, MI, bleeding, and hepatic events areadjudicated by members of an independent Clinical EventsCommittee blinded to treatment allocation. | High Risk | | Unclear | Low Risk | |
| **Attrition Bias** | | | | | | |
| *Incomplete*  *Outcome*  *data* | The primary analysis tests that ≥1edoxaban treatmentexposure is noninferior to warfarin using a pairwisecomparison (modified ITT).  Further sensitivity analyses are performed using the ITT cohortincluding all events occurring while in study and the on-treatment analytic approach based on the “per protocol principle”.  Complete information on the primary end point was ascertained for99.5% of the total 56,346 patient-years of potential follow-up.Rate of missing data (0.5%).  One patient was lost to follow-up, and 244 patients withdrew consentto follow-up; 182 of these patients had no known primary-end- pointevent and were not known to be dead. | High Risk | | Unclear | Low Risk | |
| **Incomplete Bias** | | | | | | |
| *Selective*  *reporting* | protocol available and all pre-specified outcomes are reported.  During follow-up visits, subjects are assessed for adverseevents, study end points, INR measured in a blinded fashion,and periodic safety laboratory tests (creatinine, liver function) sent to the central laboratory. | High Risk | | Unclear | Low Risk | |

**S6: Data Extraction Form**

| **NAME INVESTIGATOR FOR DATA EXTRACTION:** | |
| --- | --- |
| **STUDY ID** | |
| *Study nr*  *Name of study*  *Publication year*  *Reference* |  |
| **METHODOLOGY** | |
| *FU duration(y)*  *Number of patients* |  |
| **BASELINE**  **CHARACTERISTICS** | |
| *Age (yr)*  *CHF (n); (%)*  *CHADS ≥3 (n); (%)* |  |
| **OUTCOMES** | |
| *Stroke/Syst Embolism (SEE) (n)*  *Major bleeding (n)*  *ISTH*  *ISTH modified* |  |
